# No difference in outcomes for posterior shoulder instability surgery in patients with a normal vs. pathological radiologist reported magnetic resonance arthrogram study

**DOI:** 10.1016/j.xrrt.2026.100675

**Published:** 2026-01-28

**Authors:** Rebecca L. Byrd, Robert J. Reis, Chandler M. Catanzaro, Megan E. Welsh, Lauren E. Schell, Mitchell H. Negus, William R. Barfield, Richard J. Friedman, Josef K. Eichinger

**Affiliations:** Medical University of South Carolina, Charleston, SC, USA

**Keywords:** Posterior shoulder instability, Capsulolabral repair, Machine resonance arthrogram, Arthroscopic stabilization, Clinical examination, Patient-reported outcomes

## Abstract

**Background:**

Despite improvement in diagnostic imaging techniques, there remain patients with clinical posterior shoulder instability (PSI) with so called normal or nonpathologic findings on magnetic resonance arthrogram (MRA). This diagnostic dilemma creates challenges for clinicians and patients who fail conservative treatment when determining treatment options due to the incongruent findings on physical exam and MRA. We hypothesized that patients undergoing capsulolabral repair with a clinical exam consistent with PSI but normal radiologist reported MRA findings would experience similar clinical improvement compared to patients with PSI and pathological radiologist reported MRA findings.

**Methods:**

A database of prospectively enrolled patients was reviewed to identify patients who underwent surgery for PSI between 2016 and 2022. Inclusion criteria were a positive posterior load and shift test on examination under anesthesia, a preoperative MRA with an accessible radiologist report, and minimum 2-year follow-up. Patients with anterior or multidirectional instability on examination under anesthesia, rotator cuff lesions, a Beighton score greater than 6, glenoid bone loss or fracture, or history of prior shoulder surgery on the affected side were excluded. The interpreting radiologist's MRA impression of the posterior labrum and capsule was used to classify patients into the normal MRA (N = 16) or pathologic MRA (N = 18) cohort. Patient-reported outcome measures compared between groups included visual analog scale (VAS) pain, Single Assessment Numeric Evaluation (SANE), and Western Ontario Shoulder Instability Index.

**Results:**

Thirty-four patients with a mean age of 34.7 and body mass index of 27.7 were included in the study. Patient sex was the only significantly different demographic variable between normal MRA and pathological MRA groups (75.0% female vs. 16.7% female, *P* < .001). At the final follow-up (mean: 41.7 vs. 36.1; *P* = .361), both the normal MRA and pathological MRA groups demonstrated significant improvements in SANE (mean: 29.8, *P* < .001; mean: 41.6, *P* < .001) and reductions in VAS Pain (mean: −2.1, *P* = .005; mean: −3.2, *P* < .001). When compared between groups, there were no statistically significant differences in mean VAS Pain, SANE, or Western Ontario Shoulder Instability Index. Three patients (18.8%) in the normal MRA group required an arthroscopic capsular release for adhesive capsulitis vs. 1 patient (5.6%) in the pathological MRA group (*P* = .233).

**Conclusion:**

A careful clinical exam is the most important factor when determining indication for PSI surgery. Regardless of the radiologist interpretation, patients with symptomatic PSI can benefit from arthroscopic stabilization surgery.

The shoulder is an intricate anatomical structure that is crucial in assuring mobility and strength of the upper extremity. The shoulder is often subject to trauma, such as joint subluxation or dislocation, which can lead to labral deficiency, capsular elongation, and shoulder capsule and ligament tears.^5^ These shoulder pathologies can predispose the shoulder to the development of posterior instability, which can manifest as pain and weakness with shoulder flexion due to loss of posterior containment of the humeral head.^2^ However, there are risk factors such as ligament laxity and glenoid dysplasia or retroversion that also predispose to posterior shoulder instability.^7^

A comprehensive clinical evaluation is necessary for determining the cause of posterior instability and identification of pathologic lesions before arthroscopic surgery is considered.^4^^,^^11^^,^^15^ Diagnostic imaging is an important adjunct to confirm the diagnosis and identify the anatomical lesions responsible for instability.^6^ Although many surgeons believe a nonarthrogram magnetic resonance imaging is acceptable for determining pathological findings, magnetic resonance arthrogram (MRA) provides far more detail including identification of subtle Kim's lesions (chondrolabral separation) and allows for objective determination of pathologic capsular enlargement.^10^^,^^11^ Increased posterior capsule area on MRA has been shown to correlate with posterior shoulder instability.^6^ Galvin et al found a linear capsular measurement greater than 14 mm on axial MRA to be 96% specific for posterior shoulder instability. Despite improvement in diagnostic imaging techniques, there remain patients with clinical posterior instability with so called normal or nonpathologic findings on MRA. This diagnostic dilemma creates challenges for clinicians and patients who fail conservative treatment when determining treatment options due to the incongruent findings on physical exam and MRA.

We sought to determine the prevalence of patients who underwent arthroscopic capsulolabral repair for symptomatic posterior shoulder instability despite a MRA defined by the interpreting radiologist as having no identifiable posterior shoulder instability related findings and compare their postoperative outcomes to patients with a pathologic MRA. We hypothesized that patients undergoing capsulolabral repair with a clinical exam consistent with PSI but normal radiologist reported MRA findings would experience similar clinical improvement compared to patients with PSI and pathological radiologist reported MRA findings.

## Materials and methods

We retrospectively reviewed a prospective surgical outcomes database to identify patients who underwent arthroscopic capsulolabral repair for symptomatic recurrent posterior shoulder instability by a single fellowship-trained shoulder and elbow surgeon between August 2016 and July 2022. Institutional review board approval was granted from our institution's central institutional review board (Pro00056950). Inclusion criteria were unidirectional posterior shoulder instability on clinical exam, a positive posterior load and shift test on examination under anesthesia (EUA), a preoperative direct MRA read by a radiologist, and a minimum of 2 years follow-up. Patients were excluded if they had anterior or multidirectional instability on clinical exam or EUA, rotator cuff lesions on MRA, a Beighton score greater than 6, glenoid bone loss or fracture, or history of prior shoulder surgery on the affected side. A thorough attempt to access radiologist reads was made for all patients with an available MRA. However, in cases with an inaccessible radiologist report, it was not feasible to contact the imaging center where the MRA was performed to obtain the radiologist report. Clinical exam maneuvers to assess posterior instability included the jerk test and posterior load and shift. Prior to arthroscopic capsulolabral repair, all patients had symptomatic recurrent posterior instability on clinical exam which failed to resolve with a minimum of 12 weeks of physical therapy. The decision to undergo surgery was dependent on several factors, including the patients' preferences, pain, function, occupation, and athletic participation. Patients were classified as normal MRA (N = 16) or pathological MRA (N = 18) based on the interpreting radiologist's impression of the labrum.

Demographic variables collected preoperatively included patient age, sex, body mass index, hand dominance, and laterality. Visual analog scale (VAS) pain and Single Assessment Numeric Evaluation (SANE) were collected preoperatively and postoperatively. Western Ontario Shoulder Instability Index (WOSI) was only collected postoperatively at the final follow-up. WOSI was converted to a percentage with a score of 0 equaling 100% and a score of 2,100 equaling 0%. Patients were classified as having an excellent, good, or poor outcome at the final follow-up for their VAS pain, SANE, and WOSI. (VAS pain: excellent ≤1, good ≤2, poor >2; Single Assessment Numeric Evaluation (SANE): excellent ≥90, good ≥80, poor <80; WOSI: excellent ≥90, good ≥80, poor <80). Symptom onset was categorized as either traumatic or atraumatic. An injury was categorized as traumatic if the patient experienced an onset of pain or instability following a single event, such as contact during sports, a motor vehicle accident, fall, or weightlifting maneuver. The medical record was reviewed for all patients to record the radiologist's MRA findings, surgeon's MRA findings, and arthroscopic findings.

### Capsular measurements

MRA capsular measurements were performed preoperatively by a single fellowship-trained shoulder and elbow surgeon using our institution's radiological picture archiving and communication system. Imaging was obtained of both external and internal rotation views as described in White et al.^14^ In the axial plane, posterior capsular length was calculated by measuring the distance from the anterior aspect of the lesser tuberosity to the posterior cortex of the humeral head minus the anterior aspect of the lesser tuberosity to the deepest point of the posterior capsule.^4^ An axial posterior linear capsular measurement greater than 14 mm was considered an enlarged capsule by the lead surgeon.^6^

### Surgical technique

The surgical technique for all patients consisted of lateral decubitus positioning and repair with a minimum of 3 suture anchors. The surgical repair involved labral mobilization and repair with knotless suture anchors. In cases with a pathologically enlarged capsule, a plication of the capsule was performed. The size of the patient dictated the number of anchors used. Smaller stature patients required fewer suture anchors. The extent of the repairs and placement of anchors depended on the patient's unique capsulolabral pathology. The capsular plication bite was taken and passed around the labral tear. For patients without a labral tear but diminutive labrum or enlarged capsular volume, the suture anchor was passed around a bite of capsule and the diminutive labrum. The technique had the result of creating an enlarged labrum or bumper, a reduction in the capsular volume, and tensioning of the posterior inferior glenohumeral ligament.

### Statistical analysis

Data analysis was performed using IBM SPSS Statistics software, version 28 (IBM Corp., Armonk, NY, USA) and Microsoft Excel 2019 (Microsoft Corp., Redmond, WA, USA). A Shapiro–Wilk test revealed all variables were nonparametric. Therefore, Mann–Whitney U-tests were used to compare demographic and patient-reported outcome measures between the normal MRA and pathological MRA groups. Wilcoxon signed-rank tests were used to determine if improvement in VAS pain and SANE reached statistical significance in the normal and pathological MRA groups. An alpha value less than 0.05 indicated a statistically significant difference for all tests.

## Results

During the period between August 2016 and December 2022, 59 patients underwent posterior capsulolabral repair at our academic institution. Ten of these patients had an outside MRA with an inaccessible radiologist report for review and were excluded from further review. Four patients had a concomitant rotator cuff tear, 4 patients had a Beighton score greater than 6, and 7 patients were lost to follow-up. Therefore, 34 patients were included in the study. The interpreting radiologist reported a posterior tear in 18 (52.9%) patients and a normal labrum in 16 (47.1%) patients. Capsular pathology was not reported in any of the patients. Therefore, 18 patients were categorized as pathological MRA and 16 patients as normal MRA.

For the 34 patients included in this study, mean age was 34.7 (range: 18 to 59), 15 (44.1%) were female, the mean body mass index was 27.7 (range: 18 to 45), and the mean follow-up duration was 38.7 months (range: 24 to 65). A greater proportion of normal MRA patients were female in comparison to pathological MRA patients (75.0% vs. 16.7%, *P* < .001). No other demographic variables were significantly different between the 2 groups (Table I). Overall, 3 (8.8%) reported a dislocation prior to surgery, 25 (73.5%) had traumatic onsent of pain and/or instability, and 19 (55.9%) had surgery performed on their dominant arm.

**Table I:** Comparison of demographics and injury characteristics between the pathological MRA and normal MRA groups.

Demographics	Normal MRA (N = 16)	Pathological MRA (N = 18)	*P* value
	Mean ± SD	Mean ± SD	
Age	32.1 ± 9.8	37.1 ± 8.0	.151
BMI	26.0 ± 5.3	29.3 ± 6.1	.084
Follow-up (mo)	41.7 ± 14.9	36.1 ± 11.9	.361

	**N (%)**	**N (%)**	

Female sex	12 (75.0)	3 (16.7)	**<.001**
Traumatic symptom onset	12 (75.0)	13 (72.2)	.271
Dislocation	2 (12.5)	1 (5.6)	.476
Surgery on dominant arm	8 (50.0)	11 (61.1)	.515

*BMI*, body mass index; *MRA*, magnetic resonance arthrogram; *SD*, standard deviation.

Bold indicates statistical significance.

**Figure 1:**
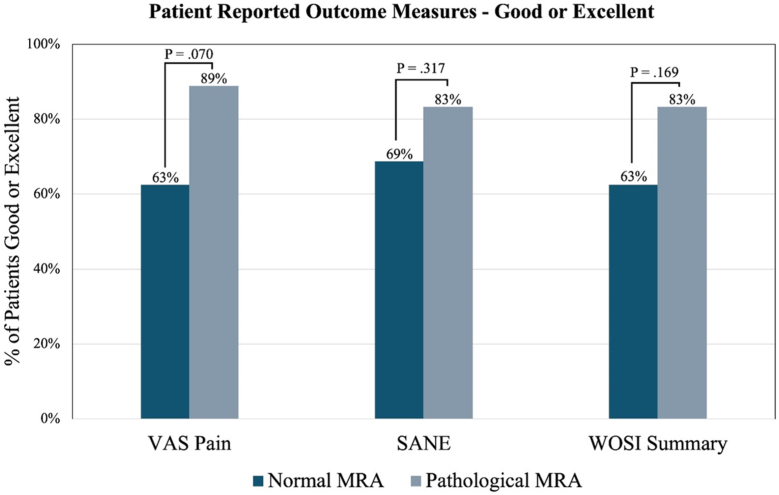
Comparison of the proportion of normal MRA vs. pathological MRA patients with a good or excellent outcome at the final follow-up. (VAS Pain: excellent ≤1, good ≤2; SANE: Excellent ≥90, Good ≥80; WOSI: Excellent ≥90, Good ≥80). *VAS*, visual analog scale; *SANE*, Single Assessment Numeric Evaluation; *WOSI*, Western Ontario Shoulder Instability Index; *MRA*, magnetic resonance arthrogram.

No statistically significant difference in outcome measures was observed preoperatively or postoperatively between normal MRA and pathological MRA groups (Table II). A greater percentage of pathological MRA patients had a good or excellent VAS pain (89% vs. 63%; *P* = .070), SANE (83% vs. 69%; *P* = .317), and WOSI Summary (83% vs. 63%; *P* = .169) at the final follow-up; however, none these differences reached statistical significance (Fig 1). Average improvement in SANE from preoperative to the last follow-up was 29.8 (range: −20 to 68; *P* < .001) and 41.6 (range: 0 to 80; *P* < .001) for the normal MRA and pathological MRA groups, respectively. Additionally, both groups had a statistically significant reduction in VAS pain, with average reported changes of −2.1 (range: −7 to 2; *P* = .005) for the normal MRA group and −3.2 (range: −7 to 1; *P* < .001) for the pathological MRA group.

**Table II:** Comparison of preoperative and postoperative patient-reported outcome measures between the normal MRA and pathological MRA groups.

Outcome measures	Normal MRA (N = 16)	Pathological MRA (N = 18)	*P* value
	Mean ± SD	Mean ± SD	
Preoperative			
VAS Pain	4.7 ± 2.1	4.6 ± 1.5	.670
SANE	47.6 ± 9.3	45.5 ± 20.8	.609
Postoperative			
VAS Pain	2.6 ± 2.9	1.4 ± 1.3	.780
SANE score	77.4 ± 26.7	87.1 ± 11.8	.676
WOSI summary	72.5 ± 30.6	86.6 ± 13.8	.309
WOSI physical symptoms	74.1 ± 29.5	88.2 ± 11.4	.379
WOSI sports recreation/work	71.3 ± 30.0	87.7 ± 11.4	.065
WOSI lifestyle	73.1 ± 33.4	86.6 ± 16.3	.717
WOSI emotions	68.8 ± 37.8	80.0 ± 20.1	.878
Change			
VAS Pain	−2.1 ± 2.6	−3.2 ± 1.8	.176
SANE	29.8 ± 28.4	41.6 ± 23.5	.194

*MRA*, magnetic resonance arthrogram; *VAS*, visual analog scale; *SANE*, Single Assessment Numeric Evaluation; *WOSI*, Western Ontario Shoulder Instability Index; *SD*, standard deviation.

A revision procedure was required for 4 of the 34 patients (11.8%). Three patients (18.8%) in the normal MRA group required revision for adhesive capsulitis vs. 1 patient (5.6%) in the pathological MRA group (*P* = .233).

Surgeon disagreement occurred for 8 (50%) of the Normal MRA patients. The surgeon disagreed with the radiologist report and believed the MRA showed a labral tear was present on MRA (Table III). Diagnostic arthroscopy revealed labral tears in 7 of these patients, and a Kim's lesion in 1. The lead surgeon was in agreement with the radiologist report for the remaining 8 (50%) Normal MRA patients and believed the labrum appeared intact on MRA. However, utilizing the method described by Galvin et al, the lead surgeon believed all had a patulous posterior capsule. Diagnostic arthroscopy revealed a labral tear in 2 of these patients, a Kim's lesion in 2, and an intact labrum in 4. The 4 patients with an intact labrum but enlarged posterior capsule underwent capsulorrhaphy only. Additionally, all 8 had an enlarged posterior capsule on arthroscopy.

**Table III:** Comparison of the interpreting radiologist's and surgeon's MRA impression with arthroscopic findings.

Patient no.	Radiologist's impression of MRA		Surgeon's impression of MRA		Arthroscopic findings	
	Labrum	Capsule	Labrum	Capsule	Labrum	Capsule
Normal MRA						
1	N	N	N	Enlarged∗	Intact	Enlarged
2	N	ND	N	Enlarged∗	Tear 9-6	Enlarged
3	N	ND	N	Enlarged∗	Intact	Enlarged
4	N	ND	N	Enlarged∗	Intact	Enlarged
5	N	ND	N	Enlarged∗	Intact	Enlarged
6	N	ND	N	Enlarged∗	Kim's lesion 5-9	Enlarged
7	N	ND	N	Enlarged∗	Kim's lesion 5-8	Enlarged
8	N	ND	N	Enlarged∗	Tear 6-10	Enlarged
9	N	ND	Posterior tear	Enlarged∗	Tear 6-10	Enlarged
10	N	ND	Posterior tear	N	Kim's lesion 4-7	N
11	N	ND	Posterior Tear	Enlarged∗	Kim's lesion 3-7	Enlarged
12	N	ND	Posterior tear	N	Tear 8-11	N
13	N	ND	Posterior tear	Enlarged∗	Tear 7-11	Enlarged
14	N	ND	Posterior tear	Enlarged∗	Tear 7-10	Enlarged
15	N	ND	Posterior tear	Enlarged∗	Tear 7-10	Enlarged
16	N	ND	SLAP tear	N	Tear 9-12	N
Pathological MRA						
17	Posterior tear	ND	Posterior tear	N	Kim's lesion 6-10	N
18	Posterior Tear	ND	Posterior tear	N	Tear 6-10	Enlarged
19	Posterior tear	ND	Posterior tear	N	Kim's lesion 6-10	N
20	Posterior tear	ND	Posterior tear	Enlarged∗	Tear 5-8	Enlarged
21	Posterior tear	ND	Posterior tear	N	Tear 6-11	N
22	Posterior tear	ND	Posterior tear	N	Tear 6-11	Enlarged
23	Posterior tear	ND	Posterior tear	Enlarged∗	Tear 7-11	Enlarged
24	Posterior tear	ND	Posterior tear	N	Tear 8-10	N
25	Posterior superior tear	ND	Posterior superior tear	N	Tear 6-10	N
26	Posterior superior tear	ND	Posterior superior tear	N	Tear 7 -10	N
27	SLAP tear	ND	SLAP tear	N	Tear 10-3	N
28	SLAP tear	N	SLAP tear	N	Tear 7-12	N
29	SLAP tear	ND	SLAP tear	Enlarged∗	Tear 7-12	Enlarged
30	SLAP Tear	ND	SLAP Tear	Enlarged∗	Tear 7-11	Enlarged
31	SLAP Tear	ND	SLAP Tear	Enlarged∗	Tear 10-2	Enlarged
32	SLAP Tear	ND	SLAP Tear	N	Tear 8-12	Enlarged
34	SLAP Tear	ND	SLAP Tear	Enlarged∗	Tear 7-11	Enlarged
33	Anterior Tear	N	Anterior tear	N	Tear 6-11	Enlarged

*N*, Normal; *ND*, Not Described in radiologist's report; *MRA*, magnetic resonance arthrogram; *SLAP*, superior labrum anterior to posterior.

∗ Posterior capsular length >14 mm on axial MRA.

The surgeon agreed with the radiologist report of the labrum for all of the Pathological MRA patients. Arthroscopy revealed a labrum tear in 14 patients and a Kim's lesion in 2. Interestingly, both the surgeon and radiologist reported an anterior labrum tear on MRA in patient number 33. This patient had a positive O'Brien's test, posterior load and shift, and anterior apprehension. However, the patient had posterior instability and no anterior instability on EUA. A posterior labrum tear extending from approximately the 6 to 11 o'clock position was present on arthroscopy.

## Discussion

The results of our study indicate that there is no significant difference in the clinical outcomes among patients who underwent capsulolabral repair for posterior instability regardless of preoperative radiological imaging results at a minimum of 2-year follow-up. However, there was a trend toward a lesser proportion of patients with a normal MRA having a good or excellent VAS pain, SANE, and WOSI at the final follow-up (Fig. 1). The revision rate trended higher in the normal MRA cohort as well. One possible explanation for these differences is that larger labrum tears may be more likely to be reported by radiologists on MRA. This may allow for a greater patient-reported outcome measure improvement and a decreased likelihood of postoperative stiffness in patients with a pathological MRA compared to those with less dramatic preoperative tissue damage. While patients with a normal reported MRA improved with instability surgery, one must be very judicious in operating on patients with subtle signs of instability.

We chose to include all superior labrum anterior to posterior (SLAP) tears in this study, and although a type 2 or 3 SLAP tear is not considered an instability finding, radiologists do not frequently specify the type of SLAP tear or if there was extension of the labral tear posteriorly. Therefore, we wanted to include these tears since some SLAP lesions extend posteriorly and are associated with posterior instability.^13^ Additionally, it is important to note that there are multiple reasons that could lead to a normal MRA read such as multidirectional instability, Kim lesion, etc. Specifically, there is a lack of specific diagnostic criteria for a Kim lesion, meaning depth of tear or irregularity.^12^ Often PSI patients do not have a labral tear but do have a diminutive labrum. If they have a small labrum, they are unlikely to have a tear, making capsular volume measurements critical.^3^ If the radiologist is unfamiliar with these measurements, they will be forced to read a MRA as normal. This is important to consider in clinical decision making when a MRA is read as normal.

Previous studies have indicated that a preoperative MRA is the gold standard in diagnosing posterior shoulder instability as it allows the best visual of intraarticular structures.^1^ However, Jonas et al discuss that MRA sensitivity in detecting labral tears is lower than previously thought indicating that MRA may have more diagnostic difficulties in diagnosing glenohumeral instability.^9^ On exam, patients are more likely to report an insidious onset of generalized shoulder pain and weakness, often lacking a history of trauma, when compared to traumatic anterior instability.^1^

The findings of this study reinforce the need for a critical shoulder exam, as well as the place of radiographical imaging.^2^ The ever-advancing field of radiology is an asset in orthopedic diagnoses; however, it cannot replace the importance of a clinical exam. In this case, and others, radiological imaging should be used to aid in the diagnosis but not to solely make the diagnosis. In addition, the results of this study also indicate the need for judicial reading of radiological imaging to ensure accurate diagnosis.

One interesting area for continued exploration that we did not assess at this time is a relationship between the prevalence of normal MRA between symptomatic males and females. From clinical experience and established studies, we know females have a greater degree of ligamentous laxity.^8^ This is often associated with increased joint instability, which can be symptomatic but have no evidence on radiographic studies. Therefore, due to this association, a next step would be to investigate if symptomatic females are more likely to have a normal MRA compared to their male counterpart, and if so, how that should influence our approach to caring for women in an orthopedic setting.

### Limitations

This study is not without its limitations. First, MRAs were not analyzed by a blinded outside surgeon, which would provide a more objective interpretation for comparison to the radiologist report. This would also allow for inter-rate agreement analysis. Additionally, The MRA reports came from a variety of imaging centers, so it was not possible to determine inter-rater and intrarater reliability. Additionally, the varying quality of MRA interpretation due to inconsistent protocols and radiologist experience may create more normal reads than is observed in other clinical practices. While a limitation of the study, we believe this has an advantage as it represents a real-world scenario in which the radiologist's report is sent to the patient and the insurance company for evaluation of adequacy of indication for surgery. This can create a difficult situation for the provider and patient as it requires additional time and effort to explain to the patient why the interpretation of the radiologist is insufficient or incorrect. Another limitation is WOSI scores were only recorded postoperatively at final follow-up. Without a preoperative WOSI score, our study lacks a baseline patient-reported measurement of instability.

## Conclusion

A thorough clinical exam is the most important factor when determining indication for shoulder instability surgery. We can conclude that regardless of the radiologist interpretation of MRA, patients with symptomatic posterior shoulder instability do benefit from arthroscopic stabilization surgery.

## Disclaimers:

Funding: No funding was disclosed by the authors.

Conflicts of interest: The authors, their immediate families, and any research foundations with which they are affiliated have not received any financial payments or other benefits from any commercial entity related to the subject of this article.
